# Natural Bioactives in Cancer Treatment and Prevention

**DOI:** 10.1155/2015/182835

**Published:** 2015-03-26

**Authors:** Yih-Shou Hsieh, Shun-Fa Yang, Gautam Sethi, Dan-Ning Hu

**Affiliations:** ^1^Department of Medical Research, Chung Shan Medical University Hospital, Taichung 402, Taiwan; ^2^Institute of Biochemistry and Biotechnology, Chung Shan Medical University, Taichung 402, Taiwan; ^3^Institute of Medicine, Chung Shan Medical University, Taichung 402, Taiwan; ^4^Department of Pharmacology, Yong Loo Lin School of Medicine, National University of Singapore, Singapore 119077; ^5^Tissue Culture Center, New York Eye and Ear Infirmary, New York Medical College, New York, NY 10009, USA

Natural bioactives are generally referred to the compounds exclusive of essential nutrients that have specific biological activity to human. From several decades ago to now, cancer continues to be the leading lethal cause worldwide. Studies have shown that natural phytochemicals derived from certain plants have the capability to prevent carcinogenesis. In this special issue, we collected numerous studies which provide novel evidence to support the opinion. For instance, epigallocatechin gallate inhibits migration of human uveal melanoma cells; marine sponge* Hyrtios* sp. extract induces apoptosis in human colorectal carcinoma RKO cells with different p53 status; Andrographolide induces apoptosis of C6 glioma cells via the ERK-p53-caspase 7-PARP pathway; and osthole induces human colon cancer cell death and inhibits migratory activity.

We also collected some review articles in this special issue. A paper evaluated the cancer therapeutic potential of cardiac glycosides. A paper proposed vitamin A as the potent anticancer agent on targeting cellular retinol binding proteins. Three other papers addressed the anticancer molecular mechanisms of betulin, Goniothalamin, and Zerumbone. In summary, it is therefore believed that the appropriate application of natural bioactives should be a supplementary and safe way that enhances the efficacy of cancer therapy.

*Yih-Shou Hsieh* *Shun-Fa Yang* *Gautam Sethi* *Dan-Ning Hu*

